# Psychological and Situational Variables Associated with Objective Knowledge on Water-Related Issues in a Northern Spanish City

**DOI:** 10.3390/ijerph18063213

**Published:** 2021-03-19

**Authors:** Elena Andrade, Gloria Seoane, Sergio Vila-Tojo, Cristina Gómez-Román, Jose-Manuel Sabucedo

**Affiliations:** Department of Social Psychology, Basic Psychology and Methodology, CRETUS Institute, University of Santiago de Compostela, 15782 Santiago de Compostela, Spain; elena.andrade@usc.es (E.A.); mgloria.seoane@usc.es (G.S.); cristina.gomez@usc.es (C.G.-R.); josemanuel.sabucedo@usc.es (J.-M.S.)

**Keywords:** water-related issues, water objective knowledge, water domestic consumption, social perception, psychological factors, urban population

## Abstract

This study brings together the level of objective knowledge on water-related issues and other variables of psychological and situational nature. A random sample of 459 participants was employed, selected proportionally based on sex and age. In this sample, knowledge on the water-related issues tended to be low, particularly related to the direct source of water in the household, the type of services involved in the management, and consumption itself. In order to understand both the relationship with knowledge on water and the relative importance of all the other factors, a regression model was formulated. The highest standardised effect was for sex, followed by occupation, political leaning, and water-related emotions. The best level of knowledge was attained if the residents were male, if they were actively employed or unemployed, if their political leaning was towards the left, and if they demonstrated greater emotional involvement with the water use. Consequently, the design of programmes would need to consider that the information flow must be greater for citizens as a whole, particularly for certain groups such as women and students. It should contribute to the realistic perception of water as a problem and to seek emotional involvement.

## 1. Introduction

Guaranteeing suitable water services in urban areas, characterised by a burgeoning residential demand, is one of the greatest challenges faced by local authorities [1]. There have been several ways in which those responsible for water management have attempted to reduce this demand in the urban setting. Technological solutions address what has been referred to as “supply management” and account for an important section of the literature [2]. However, such solutions are not always associated with low consumption [3].

Recent research emphasises a second and highly important perspective, sometimes referred to as “demand management” [2,4]. This latter approach is inspired by progress made explaining the water use behaviour rather than technological solutions or on infrastructure.

Understanding the population’s water consumption and conservation behaviour is key for the good management of the service. Additionally, this behaviour has been linked with at least three types of psychological influences: reasoned, non-reasoned, and situational [5]. Variables such as knowledge and attitudes would be reasoned influences, habits and emotions would correspond to the definition of non-reasoned influences, and family composition and education would be situational factors.

Knowledge is seen as a necessary condition for an individual’s behaviour. In line with this, many information campaigns and environmental education programmes are based on knowledge transfer [6]. Nonetheless, regarding the responsible consumption of water, understanding this chain between knowledge and action entails exploring the link thereof with the other factors [2,7,8,9].

In Spain, there are few studies on water demand from the resident’s perspective [10,11,12]. This paper presents a comprehensive investigation, which brings together knowledge on water-related issues and other important variables into one single analysis model.

The following section describes knowledge relevance and the selected associated variables, which are of a psychological (reasoned and non-reasoned) and situational nature. The methods section introduces an empirical study conducted in a northern Spanish city. This study’s ultimate aspiration is to draw meaningful implications for the effective design of education and awareness programs in the setting of water demand management.

### 1.1. Knowledge on Water-Related Issues

Knowledge should facilitate the valuation of water as a natural resource and the actions required for the treatment thereof. In the same way, individuals who act pro-environmentally are expected to be those who possess greater knowledge and appreciation of the problem [8]. According to Frick et al. [6], knowledge could nevertheless explain only around 6% of environmental behaviour. However, they add the caveat that this rate is underestimated, given that studies have not considered either the existence of different types of knowledge, or the effect of other variables of interest.

As for the type of knowledge needed, three dimensions of environmental knowledge have been identified [6]. The first could be interpreted as “knowing what” (system knowledge), the second would be “knowing how” (action-related knowledge), and the third would consist of “knowing the effect of each action, particularly in situations in which various options are possible” (effectiveness). Rather than a structure of knowledge, we could speak of a sequence, which starts with a basic understanding of the problem (system knowledge). This enables the individual to acquire the other dimensions.

Besides, knowledge on water can have several different statuses. With this assertion, we intend to say that there is, at least, an important difference between subjective and objective knowledge. Aware of this difference, Carlson et al. [13] stated that a consumer’s objective knowledge involves “*the accurate stored information or what we know; while subjective knowledge is an individual’s perception of his or her own knowledge or what we think we know*”. According to Marlow et al. [14], key aspects of objective individual knowledge would include the water cycle in the urban setting or the impact of urban development on health, as well as details related with the supply and treatment of water.

Empirical findings on the link between water objective knowledge and pro-environmental behaviour have been inconclusive. While some studies downplayed knowledge deficit strategies [15], others highlighted the association of knowledge on the water system and on how to conserve water with more conservative behaviour patterns [16,17,18]. Particularly, the latter coincide in underscoring the need to study knowledge on an individual level in connection with other variables. The prevalent notion continues to be that of incorporating processes of various types (conscious cognitive processes, non-cognitive processes, and situational factors) into psychological behaviour prediction models.

At a first stage of analysis, awareness and the way in which knowledge is acquired appear to matter [19]. Indeed, lack of awareness is one of the most important barriers for daily conservation behaviour [20]. When individuals are more involved due to interest, risk, or expediency, they will tend more towards conservation and the responsible consumption of water. The same degree of perception of the problem and personal involvement would also affect the ability to process this type of environment-related information [21] and to understand the effectiveness of water conservation actions [12].

Initiatives for water demand management rely on knowledge to enable residents to decrease their potable water consumption [22]. Shifting residents toward sustainable water consumption practices, thus, requires gradually instilling awareness and understanding of the environment and water problem.

### 1.2. Psychological Variables Associated with Water Use and Conservation

#### 1.2.1. Attitudes

Environmental attitudes have been traditionally associated with self-reported pro-environmental behaviour. Certain attitudinal components have served to predict water conservation intention and behaviour [11,23,24,25,26]. In general, research has established a connection between attitudes to water and the environment and actual water consumption [27]. Although the knowledge–attitude linkage is not always a clear one [19], jointly very positive environmental attitudes and high system, action and efficacy knowledge and awareness seem to play a predominant role in water conservation [22].

Nowadays, attitudes may be described as having preservation and utilization dimensions. Consistent with the Theory of Ecological Attitude [28], environmental movement activism (personal readiness to actively support organised action for environmental protection) would refer to preservation; while other aspects such as conservation motivated by anthropocentric concern (support for conservationism if it provides human benefits), and confidence in science and technology (confidence that science and technology can solve environmental problems) would be indicators of utilisation. The two higher-order dimensions have been identified in several cultures [29,30,31]. Nevertheless, some doubts on their discriminant validity still remain, and a global environmental-attitude rate could be a better predictor of ecological behaviour [30].

#### 1.2.2. Political Ideology

Both political affiliation and political ideology would appear to be associated with environmental awareness [32]. More environmentally orientated individuals, with pro-environmental behaviours, tend to be of a liberal ideology; while those who are less environmentally orientated, or with less pro-environmental behaviours, identify with conservative ideology, or are apathetic, politically speaking [33,34]. This trend, however, has not been fully established [35]. Accordingly, Bradbury [36] affirmed that political ideology was not a significant predictor for environmentalism, after controlling knowledge, attitudes, and various demographic characteristics.

#### 1.2.3. Emotions

As said by Carmi et al. [37], knowledge can only be transformed into action if this knowledge has an important degree of emotion. Emotions are also considered predictors of involvement with the environment, which will have both a cognitive and affective basis. By emotions, here, we refer to specific reactions, which the misuse of water may elicit in individuals. Thus, including emotion in cognitive models enhances their explicative capacity on intention and pro-environmental behaviour [38,39,40]. Among the different emotional domains (moral emotions, connection with nature, fear or anxiety owing to environmental risk), moral emotions have been capable of accounting for almost 50% of the sustainable behaviour associated with air contamination [41]. Additionally, they are considered particularly important in the setting of water [39].

### 1.3. Situational Variables Associated with the Use and Conservation of Water

Apart from the abovementioned personal variables, different socio-demographic factors may be “proxies” for knowledge and, thus, may facilitate conservation behaviours [2,9].

#### 1.3.1. Gender

Gender matters in relation to the use of water are present even from early childhood. With only a few exceptions [42], studies have shown that women usually express better attitudes and greater concern for the environment than men [25,43,44,45,46]. Nonetheless, no gender-based differences were found in water consumption or in the intention to conserve water [24,26,47]. It may well be that the effect of gender varies according to the type of environmental behaviour studied [24]. Although, according to other studies, the differences between males and females in concern for the environment, and specifically for the scarcity of water, are to be found above all on an affective level [48,49].

#### 1.3.2. Age

The findings on the relationship between age and water conservation behaviour are diverse. Lam [24] found contradictory results and in his conclusions, he speaks of the non-effect of age. Nor did Corral-Verdugo and Pinheiro [47] find any effect of age on the reported water conservation behaviour with inhabitants from two cities in Mexico. Similar results were obtained with a Spanish population [11].

Several studies [5,17,23,25,43,44] have revealed evidence in favour of older individuals in current conservation behaviour. When measuring intention, the trend could even be reversed. In turn, Fielding et al. [3] found that older residents tended to consume more, which they associated to their being at home more often and to their having adolescent children. Consequently, they suggested that perhaps the factor of interest may not have been age per se rather than stage of life they were in.

#### 1.3.3. Education

Data on the relationship between educational level and water conservation behaviour are also unclear. In some studies, those most committed to conservation were those with the highest educational level [24,44]. However, in the study by Gregory and Di Leo [5], those with the greatest number of conservation behaviours were those with the lowest educational level. Corral-Verdugo and Pinheiro [47] found educational level to have no effect on water conservation behaviour. Nor was a significant effect found by Fielding et al. [3]; although in their case, they informed of a possible overlapping between education and income level.

Moreover, a higher educational level is sometimes associated with greater knowledge. However, perhaps it is specific knowledge that should be considered and not the general measurement of education as a precursor of water conservation behaviour [2,8,23].

#### 1.3.4. Size of Household

The number of members in the household is an important contextual variable [3,5,50]. In particular, households with fewer residents were also the most environmentally committed and, consequently, more inclined to save water [5,44]. Within a numerous family, it may be more difficult to establish conservation norms, and there could also be associated physical or financial limitations.

Several studies pointed out the dynamics between individuals and communication within the household as an important factor for water consumption [2,25,51].

In two studies [22,52], which made specific mention of the composition of the household (with aspects such as the age of children), reported lower average consumption per person as the family size increased. Only in certain specific uses, such as washing clothes and the use of the toilet, would consumption be higher in more numerous families. Others found a similar trend, showing that family size correlated positively with overall consumption and negatively with per capita consumption [11,53].

#### 1.3.5. Price

Price may also be a relevant factor [54]. Its interaction with household members and composition (e.g., retirement status) has been identified in forecasting some end-use consumption categories [52]. Water consumption models are indeed very important for water resources management because they help to understand the user’s reaction to price changes [10].

Nonetheless, modifying the price can disproportionately affect low-income households, causing inequity [55]. It has been shown that this effect is not due to the price itself, but rather aspects such as knowledge on water consumption and price. Another potential explanation is that users have a better knowledge of and reaction to the total invoice than to the marginal or average price. In fact, altering water demand will be difficult if people are not aware of what they consume and pay [51,56].

From the foregoing, the literature on knowledge and other psychological and situational aspects around the water service still throws up differing results. One other obvious issue is that progress in this setting requires studies that address not one single type of factor associated with the consumption and conservation of water, but studies that attempt to be comprehensive and, within their own practical limitations, contemplate multiple related variables [3]. Focused along those lines, this study aims to determine people’s level of objective knowledge on water-related issues and to identify the psychological and situational factors related, as well as their relative importance.

With regard to psychological factors, we intend to verify whether knowledge is associated with the perception of the problem and analyse the potential effect of attitudes, political ideology, and emotions on water objective knowledge. About situational or contextual factors, we aim to specify which socio-demographic variables are most closely linked with knowledge on water-related issues.

We share the view that knowledge is a disregarded variable and that the answers to these questions will help to clarify the strengths and weaknesses of the urban population’s knowledge on the water-related issues. Specifying these aspects will be useful for the effective design of education and awareness programs and the promotion of citizen responsibility on the water demand management.

## 2. Materials and Methods

### 2.1. Participants

The sample comprised 459 participants. This was the result of a proportional random sampling based on sex and age of the registered population in a northern Spanish city. According to official data, at the time of designing this research, the population as reported by the census was N = 79,009.

The data were collected between 15 October and 15 November 2016. There had been no water restrictions in the months prior to the data collection or while conducting this study. The average litres of rainfall recorded was 115.2 L/m^2^, and the hydrological balance went from 36.1 L/m^2^ (with 9 days of rain) in October to 88 L/m^2^ (with 13 days of rain) in November. According to the report published in October 2016 by the Spanish National Statistics Institute, mean national water consumption per inhabitant was 132 L (129 in the reference region).

The city’s water management system is public, although it has been outsourced to a private company.

### 2.2. Instrument

For the data collection, we prepared a questionnaire that contained seven parts relating to the following variables of interest: knowledge, perception of the problem, estimated consumption, environmental attitudes, emotions related with water misuse, and socio-demographic data.

#### 2.2.1. Knowledge Measurement

Knowledge was measured through specific questions on the water cycle and service management. To this end, nine items were employed. These were formulated with the help of professionals (engineers and those responsible for management), who sought a balance between technical and colloquial language. An example of the type of item used was: “Domestic water comes from…” (see Table 1).

Seven items were multiple choice, one with four possible answers, and six with three possible answers, in which only one was correct. Finally, two items were open questions and addressed the price and type of services covered by the water bill. All items were encoded with 0 and 1. The score for water-related knowledge was obtained based on the number of correct responses (0–9).

#### 2.2.2. Perception of the Problem

We considered it important to ascertain to what extent water, as a natural resource, was considered to be a problem, and to compare this issue with other similar ones in terms of the nature and impact thereof on citizens’ quality of life. Five items were prepared, in which participants had to choose a value between 0 (it is not an important problem at all) and 10 (it is highly important problem). They were specifically asked about air quality, environmental noise, water service, waste collection, and waste recycling services.

#### 2.2.3. Estimated Household Water Consumption

We considered both the number of individuals who could provide data on their consumption and the perceived consumption in litres per inhabitant per day in absolute terms. We also calculated the variance between the perceived individual consumption and the official mean consumption data in the region (129 L/m^2^).

#### 2.2.4. Attitudes

Nine items adapted to Spanish were employed from the Environmental Attitudes Inventory [30]. The items represented factors labelled environmental movement activism, conservation motivated by anthropocentric concern, and confidence in science and technology. For practical purposes, and with the reliability of responses in mind, the same scale of 0 (totally disagree) to 10 (totally agree) was maintained. Two items were formulated in the opposite sense and were re-encoded. The score for attitude for each participant was obtained through the mean of the responses in all items.

#### 2.2.5. Emotions

The emotions associated with an irresponsible use of water are also indicators of awareness raising in this issue. We used six items from the Rating Scale of Emotions towards Water Wastage [39]. Once again, the respondents assessed to what extend certain feelings related with the misuse of water described them. Examples of items were “I feel bad when I see water being wasted from a water leak in the street”, and “It bothers me when someone stays in the shower for too long”. To respond, they selected a number between 0 (does not describe me at all) and 10 (describes me perfectly). The individual score for emotion was obtained through the mean of their responses.

#### 2.2.6. Socio-Demographic Data

The habitual variables were recorded, including sex, age, marital status, highest educational level reached (with four values: basic education, baccalaureate, professional training, and university degree), current professional status (with four categories: unemployed, studying, working, and retired), and the number of individuals living in a household.

Participants were also asked to indicate which political option best represented their ideas. The item was expressed as is habitual in studies from the Spanish Centre for Sociological Research (Rey, 2004) in a graded scale between 1 (left) and 10 (right). Thus, a higher value indicated a greater degree of conservatism.

### 2.3. Procedure

The data were collected in situ, visiting the participants’ homes. This task was conducted by seven psychology graduates who had previously been instructed in a common training session.

The research was conducted respecting the rights of participants, who signed an informed consent form. Through verbal and written instructions in the questionnaire, the confidentiality of their responses was guaranteed. They were informed of the aim of the study. They were asked for sincerity, and they were offered a card with contact telephone numbers and e-mail addresses to receive additional information.

All participants had their habitual residence within the city, and only one questionnaire was completed per household.

### 2.4. Analysis

The initial description and contrasting of statistical hypotheses were performed using the *IBM SPSS Statistics* (International Business Machines Corporation. Armonk, NY, USA) package. The data were examined using analysis of variance and regression models with both quantitative and categorical variables. All variables were introduced as predictors in a linear regression model. Multicategorical variables were used as predictors after dummy coding. With dummy coding, g—1 indicator variables containing either a zero or one represented which of the g groups (e.g., occupation groups) each case belonged in. The input of the model was determined by the significant change in F and R^2^, which is by a significant consistent improvement of the explained variance. The relative importance of the variables was established by standardised regression coefficients. The main effects and possible interactions were studied. The collinearity diagnostics and studentized residuals were estimated for guaranteeing the results understanding.

## 3. Results

### 3.1. Initial Description

Of the 459 participants, 248 (54%) were female. The sample’s age was between 18 and 85, with a global mean of 48.46 (*SD* = 17.40).

Secondary and higher education had been completed by 80.8% of the participants; 52.9% were actively employed. Participants who were married (48.8%) or single (40.1%) accounted for 88.9% of the sample. The number of individuals in the household ranged between 1 and 6; nonetheless, the greater percentage corresponded to households where the number of members in the family unit is equal to 2 (32.5%), 3 (29.8%), and 4 (20.5%).

### 3.2. Knowledge on Water-Related Issues

**Figure 1:**
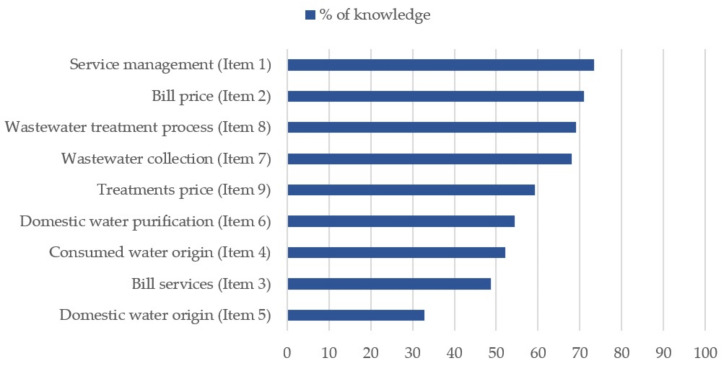
Knowledge on water-related issues by the percentage of correct answers.

On a scale from 0 to 9, mean knowledge was 5.17 (*SD* = 1.76). This figure is the equivalent of 57.49% correct answers. Figure 1 shows the percentage of knowledge achieved for each statement.

The lowest level of knowledge in the sample was related with the direct source of the water for domestic consumption, with only 32.8% correct answers. For all other specific matters regarding the water service management, correct answers ranged between 52.1% (for the question on the general origin of the city’s water) and 73.4% (regarding the type of mixed management existing). Of the total sample, 29% of participants (N = 459) were unable to specify the amount they paid for their water bills, and 51.3% had problems specifying which services or items this bill included.

### 3.3. Knowledge, Perception of the Problem, and Estimated Consumption

Descriptive statistics can be found in Table 2. Of the different environmental aspects (air quality, environmental noise, water service, waste collection, waste recycling), the highest measurements about the importance of the problem were for waste recycling and for environmental noise. Additionally, the lowest mean was for the water service.

The perception of the problem was uniform and relatively low in the sample as a whole; it was not significantly related with the level of general awareness that individuals have of the water cycle and the management conducted (Pearson correlation *r* = 0.007, *p* = 0.874; *n* = 458).

The mean perceived consumption for the sample was 157.62. The mean deviation for the official consumption data was 28.62. We found no significant correlations between knowledge and perceived consumption (*r* = −0.036, *p* = 473; *df* = 394).

### 3.4. Knowledge and Socio-Demographic Variables

All variables were introduced as predictors in a linear regression model. The significant change in *F* and *R^2^* only came about when introducing the variables of sex and occupation (see Table 3). The variables of age, marital status, educational level, and number of members in the household had no significant effects. We found no interactions between variables.

By groups, males demonstrated significantly higher knowledge. Individuals who were unemployed or retired did not demonstrate a significantly different knowledge from active workers, but did so with students. A posteriori comparison between means confirms that there was no difference between active workers (5.427) and unemployed individuals (5.707), and that it is these that have the greatest knowledge regarding water. Students (4.611) demonstrated significantly lower knowledge. Retired individuals were in an intermediate position, with a score (5) slightly higher than students and lower than the employed workers and the unemployed. However, the difference about other occupational categories did not reach significance.

### 3.5. Knowledge, Political Ideology, Attitudes, and Emotions

In order to verify both the relationship with knowledge of water and the relative importance thereof, the variables of political tendency, environmental attitudes, and emotions related with the misuse of water were incorporated. Although emotion and attitude would seem to be significantly related, in subsequent tests on the model, altering the order of introducing the variables, we have been able to verify that only emotions contributed to significantly improving the explained variance.

In Table 3, it can be seen that political leaning and emotions were significant. In this case, the higher the level of conservatism, the lower the knowledge about water, and the higher the level of emotion related with the misuse of water, the greater the knowledge.

We did not obtain significant interactions between these variables, or between demographic and personal variables. On the other hand, none of the three variables in this point (political ideology, attitudes, and emotions) presented a significant relationship with the consumption reported by the sample.

The global regression model was estimated with data from the 435 participants. The R^2^ value for the predictors as a whole was 0.29, the adjusted value thereof being 0.07. The effect size was, thus, low, as were the coefficients of the variables. Listed in order of importance, the highest standardised coefficient was for sex, followed by occupation, and with a highly similar effect, political leaning and water-related emotions.

The collinearity diagnostics (e.g., tolerance values between 0.871 and 0.947) revealed no difficulties for the interpretation of the analysis. Nor did the analysis of the residuals reveal any anomalies or influencing values (eliminated studentised residual between −2.91 and 2.49).

## 4. Discussion

The study aimed to determine the level of objective knowledge on water among the population and to identify the associated psychological and contextual factors.

### 4.1. Knowledge on Water-Related Issues

Knowledge of specific aspects of the cycle, in particular, of the direct origin of the water in households, were at low values, equivalent to those found on general competence regarding the environment among Israeli university students [37]. Another critical aspect was the information on the different services included in the water bill. The best scores were obtained when asking about the type of management (public, private, or mixed). Nonetheless, a percentage of the sample was still unable to provide data on the price they had to pay for the water and on their daily consumption. On average, however, knowledge values on the system were very similar to those obtained by Dean et al. [17] in a sample of the Australian population.

### 4.2. Psychological Variables

Consistent with Carmi et al. [37], we found a relationship between knowledge and emotions. However, none of these aspects has been significantly associated with the consumption reported. This result could be interpreted in at least two different ways. On one hand, the consumption values (owing to their high variability and the variance regarding the data from the official statistics) reflect a substantial deficit in the participants’ knowledge. A certain degree of difficulty in estimating one’s own water consumption has also been found in other cultures. North Americans tend to underestimate both the average quantity of water used in their environment, as well as their own use [57,58].

On the other hand, even when treating the mean value for perceived consumption with caution, knowledge and emotion will not suffice to explain water consumption. From our perspective, the lack of “awareness” would seem to play an important role.

Dolnicar et al. [59] proved empirically that knowledge on the water source was a key factor in their study on the consumption of desalinated and recycled water, but so too was the perception of the problem of water shortage. This awareness of the problem will, of course, be related with previous experience of difficulties in service quality and supply.

In our sample, the level of perception of the problem was low. Research into water-related aspects has frequently been conducted in populations subject to restrictions, principally in the United States and Australia [17,59,60]. Additionally, even in an a priori more environmentally aware society, the aspects of supply and treatment may be “invisible” or not perceived as being priorities [61].

As regards the role of attitudes and emotions, although there is co-variation in the score for both aspects, in our study, only emotions are significantly associated with knowledge on water. In the same way that there is prior research that addresses the relevance of emotions [37,39], there is transcultural evidence on the lower predictive capacity of attitude as opposed to knowledge in relation to sustainable behaviour [57,62].

Here, the specificity of the measurements may be a key element [8]. In this work, we use a measurement of emotions aimed at water misuse [39]. Nonetheless, we adhere to the widespread practice of employing a measurement of general attitudes towards the environment [30]. Attari [57] resorted to the New Ecological Paradigm, while Vicente-Molina et al. [62] developed their own instrument.

One further element to be considered is that citizens’ decisions regarding water are going to be affected less by attitudinal variables when they allude to fixed consumption (such as the daily shower, washing clothes, etc.) than when asked about their discretional use of water [22].

With reference to political ideology, there would seem to be consensus insofar, as this variable and political affiliation are linked to environmental awareness [32]. Our results point in the same direction as other recent studies, which associate liberalism with intrinsic motivation towards the environment [34].

### 4.3. Situational Variables

Addressing the demographic variables, we recorded a higher level of objective knowledge among males and in individuals who were unemployed or employed workers.

Some authors stressed the importance of gender and foresaw that females would show a more positive orientation towards the environment than males, in both developing and developed nations [62]. In contrast with this prediction, the results of our study reproduce those of Attari [57], who informed of a higher and more precise level of knowledge among males.

Although occupation has been mentioned as a variable of interest in the setting of household energy saving or the consumption of ecological food [63], few studies have stressed the individual’s professional status as a determining factor. Dean et al. [16] noted that the effect of involvement with the community on knowledge on water was moderated by employment. In their study, participants were divided into two large groups: employed and unemployed. Curiously, the data on the unemployed revealed a greater link between involvement with the community and knowledge of water than those of interviewees in other professional situations.

With respect to age, the findings were, thus, along the lines of the reports on water conservation with a Mexican population [47] and with a Spanish population [11].

Several studies had played down the importance of the educational level in relation to the conservation behaviour [3,47,64]. Considering the results, one can suppose that knowledge level and educational level do not represent the same thing and should be considered as two characteristics in their own right.

Moreover, the literature seems to display a certain agreement about a positive effect in favour of households with a low number of residents [3]. Our findings do not support this idea.

That shown by the socio-demographic variables contrasts with Dean et al. [17,43], who affirmed that greater knowledge corresponded with greater age and higher educational level in non-urban areas. We must undoubtedly consider both cultural differences and the fact that the aforesaid samples were broader and more heterogeneous.

The greater relative weight of contextual variables over individual ones in determined behaviours (energy consumption and others) has been repeated in the empirical research [64,65]. What is more, in accordance with Jorgensen et al. [54], the correspondence between motivational factors and behaviour in the household should arise above all in single-person households.

To expand on this notion that the estimated consumption appears to be independent of personal variables and knowledge is linked principally to characteristics of the individual with a high degree of involvement or affectation can be justified by the level of analysis we envisage. Individual and household could be understood as different levels and would, thus, be less consistent between themselves.

Until now we have, above all, stressed those aspects that this study has in common with previous research. We have also provided our perspective on certain findings. This report will be one of the scant references in our setting on knowledge related with water and associated variables. It was intended to be comprehensive, incorporating different types of factors of interest into one single model of interpreting reality. Additionally, it has merits, such is the fact that the information was recorded in situ, by motivated researchers with suitable prior data collection training. Notwithstanding, this project entailed certain conditioning factors, some of which were simply of a practical nature, which we must mention.

### 4.4. Limitations and Future Directions

The reference population was local, and the results must be extrapolated to other contexts with caution. They may indeed serve to design a larger scale study, improving the representativeness of the sample and other methodological and conceptual characteristics.

From a methodological perspective, our research has relied exclusively in the information obtained based on self-reporting.

On a conceptual level, we have focused on measuring objective knowledge, and we have placed less emphasis on forms of subjective knowledge, which have been supported in previous studies [6,37].

Similarly, the measurement of emotions selected highlights moral emotion, at the expense of other also relevant dimensions such as “emotional affinity” and “ecological fear” [41].

Finally, at the risk of obtaining a scantily operative model, we feel it necessary to incorporate variables that would accentuate the social factor, both within the household [25,51] and in relation to the setting. Variables such as social identity [25,66] would be important in a city with a predominance of the services sector and a high percentage of non-owners in housing. In turn, demographic variables, such as length of residence in the city and income level, could help us to better understand the reality regarding knowledge on water.

There is no doubt that aforementioned variables are tied to the prevailing social context, available technology, and local climate patterns. Thus, the comparison between regions with different water scarcity conditions would be necessary. At the time when the study was conducted, the population had not experienced water related difficulties. It is, thus, logical that the perception of the problem and personal involvement are low. Notwithstanding, it is of great interest to consider the profile of citizens when water is not perceived as a problem. This will no doubt help to design adapted education campaigns and programmes.

## 5. Conclusions

In an immediate manner, our study reveals that knowledge on the water-related issues tends to be low, evidently so with regard to the direct origin of the water in the household, the type of actions and services involved in this management, and, no less importantly, regarding consumption itself. It is associated with variables relating to the individual, such as gender, occupation, political tendency, and emotional involvement with the misuse of water.

Thus, the possibilities of holding a greater level of knowledge are maximised if the residents are male, if they were actively employed or unemployed, if their political leaning is towards the left, and if they demonstrate greater emotional involvement with the use of water.

Consequently, the information flow must be greater for its citizens as a whole and, in particular, for certain groups, namely females and students. The design of programmes will need to consider, in particular, the perception of water as a problem and to seek emotional involvement.

Along with Dean et al. [17], we share the notion that knowledge is a variable that has been overlooked, and it must not be taken for granted. That being said, we must also recognise that this is a highly complex issue and that the design of information campaigns based on knowledge and awareness of the problem is not always conducive to action [15]. For that reason, more than one policy for promoting water conservation would be needed [51,67].

## Figures and Tables

**Table 1:** Knowledge statements, options, and correct answers.

Knowledge Statements	Options and Correct Answers (in Bold)
1. [City name]’s water service is managed by…	a. City council b. **City council, which grants it to a company as a concession**. c. Private company
2. How much do you pay on your water bill?	Open Question
3. The water bill covers… (at least one service).	Open Question
4. Consumed water in [city name] comes from…	a. [**River/source of water**] b. [River/source of water] c. [River/source of water] d. **Springs**
5. Domestic water comes from…	a. Wastewater treatment plant b. Purification treatment plant c. **Storage tanks**
6. Once the water is captured…	a. It is stored in tanks, and then goes to the distribution network b. It is sent to purification treatment plant, where it is treated for consumption and then goes to the distribution network c. **It is sent to the purification treatment plant, where it is treated for consumption, stored in tanks and then goes to the distribution network**
7. Once the water has been used in your home, it is collected to send it…	a. Into the river b. **To a wastewater treatment plant** c. To a water treatment plant to be purified and then reuse it
8. After passing through the sewer…	a. The wastewater it is storage in a storm tanks and then it is discharged into the river b. **The wastewater is sent to a treatment plant to remove organic matter and then it is discharged into the river** c. The wastewater is sent to a treatment plant to remove organic matter and then it is sent to a purification plant
9. It is more expensive…	a. Water purification than wastewater treatment b. **Wastewater treatment than domestic water purification** c. The cost is roughly the same

Options and Correct Answers (in Bold).

**Table 2:** Descriptive statistics of perception of the problem and perceived consumption.

Variable	*M*	*Mdn*	*Mo*	*SD*
Perception of the problem				
Air quality	5.33	5.00	5	3.05
Environmental noise	6.16	7.00	8	2.72
Water service	5.14	5.00	5	3.10
Waste collection	5.73	6.00	7	3.00
Waste recycling	6.21	6.50	10	2.77
Perceived consumption	157.62	60	100	577.63

Note. *M* = mean; *Mdn* = median; *Mo* = mode; *SD* = standard deviation.

**Table 3:** Regression model for knowledge on variables of a demographic and personal type.

Variable Type	*F*	*Beta*	*t*	*p*
Demographic variables				
Gender: Female	13.828	−0.196	−4.131	0.000
Occupation	7.094			
Occupation: Unemployed		0.057	1.211	0.227
Occupation: Student		−0.156	−3.227	0.001
Occupation: Retired		−0.026	−0.534	0.594
Personal variables				
Political conservatism	6.884	−0.105	−2.164	0.031
Water-related emotions	6.560	0.103	2.155	0.032

Note. *F* = Fishers’ F; *t* = Student’s t; *p* = probability.

## Data Availability

Not applicable.
